# Do Motives to Undertake Physical Activity Relate to Physical Activity in Adolescent Boys and Girls?

**DOI:** 10.3390/ijerph120707656

**Published:** 2015-07-08

**Authors:** Jaroslava Kopcakova, Zuzana Dankulincova Veselska, Andrea Madarasova Geckova, Michal Kalman, Jitse P. van Dijk, Sijmen A. Reijneveld

**Affiliations:** 1 Institute of Health Psychology, Medical Faculty, Safarik University, Tr. SNP 1, Kosice 04011, Slovakia; E-Mails: jaroslava.kopcakova@upjs.sk (J.K.); zuzana.veselska@upjs.sk (Z.D.V.); geckova.andrea.madarasova@upjs.sk (A.M.G.); 2 Institute of Active Living, Faculty of Physical Culture, Palacky University in Olomouc, Tr. Miru 115, Olomouc 77111, Czech Republic; E-Mail: michal.kalman@upol.cz; 3 Graduate School Kosice Institute for Society and Health, Medical Faculty, Safarik University, Tr. SNP 1, Kosice 04011, Slovakia; E-Mail: j.p.van.dijk@umcg.nl; 4 Olomouc University Social Health Institute, Palacky University Olomouc, Tr. Miru 115, Olomouc 77111, Czech Republic; 5 Department of Community & Occupational Health, University Medical Center Groningen, University of Groningen, A. Deusinglaan 1, 9713 AV Groningen, The Netherlands; E-Mail: s.a.reijneveld@umcg.nl

**Keywords:** physical activity, motives for physical activity, adolescents, gender

## Abstract

Low levels of physical activity (PA) during adolescence contribute to obesity and poor health outcomes in adolescence, and these associations endure into adulthood. The aim of this study was to assess the associations between motives for PA and the level of PA among adolescent boys and girls. We obtained data regarding motives for PA and frequency of PA in 2010 via the Health Behavior in School-aged Children cross-sectional study in the Czech and Slovak Republics (n = 9018, mean age = 13.6, 49% boys). Respondents answered questions about their motives for PA and the frequency of their PA. Motives for PA were assessed using 13 items, which were structured in four groups. We explored the association between the motives for PA and sufficient PA using univariate and multivariate logistic regression models adjusted for age, and separately for boys and girls. “Good child” motives and Achievement motives were significantly associated with sufficient PA among both boys and girls. Health motives were associated with sufficient PA only among boys, and Social motives were associated with sufficient PA only among girls. Motives for PA were associated with the level of PA, and this association was partially gender dependent. These gender differences should be considered in interventions focusing on enhancement of PA.

## 1. Introduction

Developing and maintaining regular physical activity (PA) during adolescence contributes to health. Regular PA leads to physical and mental health benefits, including improvement of the physical and mental quality of life [1,2,3], and may also improve academic and cognitive performance [4]. PA plays an important role in the establishment, enjoyment and maintenance of social relationships; it provides a direct benefit by contributing to physical appearance through increased fitness, strength and weight control [5]. Low levels of PA during adolescence contribute to obesity and poor health outcomes in adolescence [1,4], and these associations endure into adulthood [6]. 

Motivation is a personal characteristic that may be one of the key factors for understanding why some people are physically active in their leisure time [7]. Motivation as a central point of the Self-Determination Theory (SDT) is mostly explored in terms of intrinsic *versus* extrinsic motivation. Intrinsic motivation is completely self-determined and is reflected in behavior performed for the pleasure in and stimulation of the activity itself [8]. More intrinsic, self-determined forms of motivation are associated with optimal functioning and well-being [8]. Regarding PA, adolescents who were intrinsically motivated were more likely to be physically active [9]. The Goal Contents Theory (GCT), a theory belonging to the SDT field, does not only distinguish between intrinsic and extrinsic goals and their impact on motivation and wellness. The GCT more specifically outlines extrinsic goals, such as financial success, appearance, and popularity/fame, contrasting these with intrinsic goals, such as community, close relationships, and personal growth, with the former more likely to be associated with lower wellness and greater ill-being [8,10]. 

From the SDT theoretical background we used GCT as the basis to cluster the motivation for PA. Regarding SDT, several validation studies have been published on the clustering of motives, e.g., [11,12,13,14]. The GCT provides another framing for the clustering; some of these clusters are clearly extrinsic (e.g., achievement), but some are combination of intrinsic and extrinsic motives (e.g., social, health, “Good child” motives) [14].”

The amount of PA and motives for PA may differ highly by gender [7,13,15,16] and by age [16]. Frequently reported motives among both genders were health and enjoyment [7,16,17]. Friendship and competition were relatively important motives for boys, and they appeared much less important for girls [16,18,19]. In contrast, losing weight was a relatively common motive in girls but not in boys [20]. For adolescent girls in particular, the pressure to conform to social stereotypes is a key motivator [17]. Adolescent boys more often reported intrinsic motives, while girls more often reported extrinsic motives [13,16]. Gillison *et al*. [20] indicate in their study about participation of boys in PA that it forms a large part of their social life and is accepted as something that they would be prepared to do in order to spend time with their friends. Conversely, for girls it was notably separate from their social lives [20].

Understanding why adolescents are physically active or inactive could significantly contribute to the design and delivery of health-promoting interventions. There are several motives and factors that influence whether or not boys and girls participate in PA [13,14,21]. Generally, the older the adolescents are, the less physically active they are [22,23,24]. However, inconsistent findings are found when comparing cohorts of Slovak and Czech boys and girls over the last decade. A steep decrease of PA after 2002 was followed by a slight increase in 2010 in the Czech republic and 2014 in Slovakia among younger adolescents, characterized by an inconsistent pattern in boys, particularly in older age groups [22,23,25,26,27,28]. One of the reasons for gender differences in these trends in the prevalence of PA might be that health-promoting interventions fit better with the motives for PA of girls than of boys. Evidence on these motives and gender differences in them may thus be very useful. 

In the present study, we focus on the connection between the motives for PA and the level of PA in adolescence and on potential gender-related differences. The aim of this study is to assess the associations between motives and level of PA among boys and girls.

## 2. Methods 

### 2.1. Sample and Procedure

We used data from the Health Behavior in School-aged Children (HBSC) cross-sectional study conducted in May–June 2010 in the Czech and Slovak Republics. The HBSC is an international, school-based study conducted in collaboration with the World Health Organization, focusing on the health and health-related behavior of 11-, 13- and 15-year-old school children in their social context. More detailed information about the HBSC methodology can be found in a paper by Roberts *et al.* [29].

*Czech sample:* From a list of schools based on information from the Institute for Information on Education, a contributory organization of the Ministry of Education, Youth and Sport, 91 schools from all 14 regions of the Czech Republic were randomly chosen to create a representative sample. We contacted 91 schools, and 86 schools took part in our survey (school response rate 94.5%). According to the protocol of the HBSC study, classes from the 5th to 9th grades were selected randomly, one from each grade per school. We obtained data from 5284 adolescents from the 5th, 7th and 9th grade of elementary schools in the Czech Republic (response: 87.0%). The HBSC protocol indicates that only 11-, 13- and 15-year-old adolescents should be included, leading to the exclusion of some pupils. Therefore, the final sample consisted of 4404 Czech pupils (mean age = 13.49, 48.0% boys). 

*Slovak sample:* From a list of schools based on information from the Slovak Institute of Information and Prognosis for Education, 134 larger and smaller schools located in rural, as well as in urban, areas from all regions of Slovakia were randomly chosen to create a representative sample. We contacted 108 schools, and 106 schools took part in our survey (school response rate 98.1%). According to the protocol of the HBSC study, classes from the 5th to 9th grades were selected randomly, one from each grade per school. We obtained data from 8491 adolescents from the 5^th^ to 9^th^ grade of elementary schools in Slovakia (response: 79.5%). Non-response was primarily due to illness (10.3%) and parental disapproval of the participation of their children (7.4%). Exclusion of those outside the required age-categories led to a final sample consisting of 4614 Slovak pupils (mean age = 13.67, 48.0% boys).

The study was approved by the Ethics Committee of the Faculty of Medicine at the P.J. Safarik University in Kosice, Slovakia. According to Czech legislation the study did not have to be approved by an Ethics Committee, because it consisted of an anonymous questionnaire. Parents were informed about the study via the school administration and could opt out if they disagreed with their child’s participation in the study. Participation in the study was fully voluntary and anonymous with no explicit incentives provided for participation. Questionnaires were administered by trained research assistants in the absence of a teacher during regular class time.

### 2.2. Measures

Demographic data (age, gender) were collected using single questions and validated in the Health Behavior in School-aged Children (HBSC) surveys [26,27].

We asked regarding PA (HBSC): Over the past 7 days, on how many days were you physically active for total of at least 60 minutes per day? [26,27]. This item was developed by Prochaska *et al.* [30] to produce a reliable and valid screening measure of moderate to vigorous physical activity of children and adolescents. To assure that respondents will consider the whole variety of physical activity and will also take into account the intensity item is associated with the following introductory instruction: “Physical activity is any activity that increases your heart rate and makes you get out of breath some of the time. Physical activity can be done in sports, school activities, playing with friends, or walking to school. Some examples of physical activity are running, brisk walking, rollerblading, biking, dancing, skateboarding, swimming, soccer, basketball or skiing.” We used the global WHO recommendation for PA and we dichotomized the respondents into those with sufficient PA (7 days) and those without (0–6 days) [4,31,32].

The *motives for PA* were assessed using a question from the HBSC study consisting of 13 items examining why young people undertake leisure-time PA [21]: Here is a list of reasons that some young people give for taking part in PA in their free time. For each motive please tick how important it is for you, with as categories (1) very important; (2) fairly important; (3) not important; for the following motives (a) to have fun; (b) to be good at sport; (c) to win; (d) to make new friends; (e) to improve my health; (f) to see my friends; (g) to get in good shape; (h) to look good; (i) I enjoy the feeling of using my body; (j) to please my parents; (k) to be cool; (l) to control my weight; and (m) it is exciting. The question was first used as part of an optional PA package in the 1985/86 HBSC survey consisting of 11 items [21]. Two additional sub-items were added in the 2005/06 HBSC survey, covering weight loss and excitement. For the purpose of this study motives were divided into the four components based on the factor analyses done by Kalman *et al*. [14] with a similar purpose as the SDT of Deci and Ryan [8,10]. Items a, f, k, d and m loaded on component 1, which was labeled as *Social motives*; items g, h and l loaded on component 2, labeled as *Health motives*; items e, i and j loaded on component 3, labeled as “Good child” motives, and items b and c loaded on component 4, labeled as Achievement motives. The “Good child” motives are a combination of internal and external motivations according to GCT and SDT [8]. The higher adolescents scored in each of the motives, the higher the levels of motivation for PA that they reported. 

### 2.3. Statistical Analyses

As differences between the Slovak and Czech Republics were negligible regarding age, gender, PA and also motives for PA, the analyses were performed in merged samples. Though data were collected in two countries, it is important to indicate that the Czech and Slovak Republics were together as Czechoslovakia until 1993, when these countries separated. Due to the relatively short separation and the long common history, these two countries still share a very similar cultural and linguistic background, making differences in the findings in this regard rather unlikely [33]. We first described the characteristics of the sample by gender, using chi-square and t-tests to assess gender differences. In the next step we used crude and adjusted binary logistic regression, adjusted for age separately for boys and girls, to explore associations between motives for PA (social motives, health motives, “Good child” motives, achievement motives) and sufficient PA (on 7 days per week *vs*. less) among adolescents. In the first model we assessed the crude associations with PA of all groups of motives, and in the second model we assessed the joint associations of groups of motives with PA. The z-scores of latent variables from factor analysis (median = 0, range from −1 to 1) are herein presented. All analyses were performed using IBM SPSS 20 for Windows (IBM Corp. Released 2011). 

**Table 1:** Descriptive statistics for country, age, sufficient PA and motives for PA for the whole sample and separately for boys and girls.

	Total	Gender		*p*-Value
		Boys	Girls	
	(n = 9018)	(n = 4360)	(n = 4654)	
**Country: *n* (%) **				ns
** Slovakia**	4614 (51.2)	2225 (51.0)	2385 (51.2)	
**Czech republic**	4404 (48.8)	2135 (49.0)	2269 (48.8)	
**Age: *n* (%)**				<0.01 ^a^
** 11 years old**	2687 (29.8)	1315 (30.2)	1370 (29.4)	
** 13 years old**	3204 (35.5)	1500 (34.4)	1702 (36.6)	
** 15 years old**	3127 (35.5)	1545 (35.4)	1582 (34.0)	
**Sufficient PA: *n* (%)**	2006 (22.2)	1211 (28.2)	795 (17.3)	<0.001 ^a^
**Motives for PA: Mean (SD)**				
** Social motives**	4.49 (0.46)	4.94 (1.01)	5.05 (0.99)	<0.001 ^b^
** Health motives**	4.80 (1.07)	4.92 (0.98)	5.08 (1.01)	<0.0001 ^b^
** “Good child” motives**	4.68 (0.94)	5.00 (1.02)	5.00 (0.98)	<0.0001 ^b^
** Achievement motives**	4.66 (1.09)	5.30 (1.00)	4.72 (0.91)	<0.0001 ^b^

SD—standard deviation. ^a^ Chi-square test; ^b^ t-test.

## 3. Results and Discussion

### 3.1. Results

The background characteristics of the sample are presented in Table 1. Of the whole study sample 22.2% reported sufficient PA, 17.3% for girls and 28.2% for boys. Boys reported significantly more PA on 7 days/week compared with girls. Girls in comparison to boys scored significantly higher in social and health motives, while boys scored higher in “Good child” and achievement motives. 

In the next step we used crude and adjusted binary logistic regression analyses, adjusted for age separately for boys and girls (Table 2). The results, as shown in Table 2, indicate that all motives for PA were associated with PA, after adjustment for differences in age. With mutual adjustment, “Good child” motives and Achievement motives were significantly associated with sufficient PA among both boys and girls. Health motives were associated with sufficient PA only among boys, and Social motives were associated with sufficient PA only among girls. 

**Table 2:** Associations between motives for PA and sufficient PA (on 7 days per week) adjusted for age: Odds ratios (OR) and 95% confidence intervals (95% CI) from binary logistic regression (crude and adjusted) by gender.

	Univariable		Adjusted for other Motives	
	Boys OR (95% CI)	Girls OR (95% CI)	Boys OR (95% CI)	Girls OR (95% CI)
**Social motives**	1.10 (1.03–1.18) ^**^	1.16 (1.07–1.26) ^***^	1.06 (0.99–1.14) ^ns^	1.17 (1.08–1.28) ^***^
**Health motives**	1.23 (1.14–1.32) ^***^	1.06 (0.98–1.15) ^ns^	1.17 (1.09–1.26) ^***^	1.08 (1.00–1.17) ^ns^
**“Good child” motives**	1.47 (1.36–1.58) ^***^	1.24 (1.13–1.35) ^***^	1.47 (1.36–1.59) ^***^	1.26 (1.16–1.38) ^***^
**Achievement motives**	1.49 (1.39–1.60) ^***^	1.42 (1.30–1.54) ^***^	1.50 (1.39–1.61) ^***^	1.42 (1.30–1.55) ^***^

^***^ *p* < 0.001; ** *p* < 0.01; ^ns^—not significant.

### 3.2. Discussion

We explored the associations between motives of PA and the level of PA separately for boys and girls. Our results indicate that associations between motives and the level of PA partially differ by gender. We found that “Good child” motives and achievement motives are associated with the level of PA among boys, and also among girls. Health motives were associated with sufficient PA only among boys, and Social motives were associated with sufficient PA only among girls. 

Our study shows that for both boys and girls “Good child” motives and achievement motives were associated with sufficient PA; however, they were rated higher by boys. In other studies [13,21] boys also reported higher rates of achievement motivation than girls, and the association of achievement motives with PA was confirmed in both boys and girls [13], which is in line with our findings. An explanation for this finding may be that a perception of success, e.g., winning, being good in sport or parental approval, is a very important motivator for adolescent PA, particularly in Europe and North America, as well [13]. If causal, this may direct the design of interventions to improve PA greatly. The achievement and competition element should then be enhanced. However, this runs the risk of mostly attracting those who are good in PA [17,18]. The balance between this disadvantage and the potential advantages evidently requires further research.

Although girls rated health motives higher than boys, the association of health motives with the engagement in PA was found only among boys. Several other studies provided no consistent evidence for gender differences in health motives, e.g., [15,21,34]. In contrast to our findings, the study of Iannotti *et al*. [13] found that a higher importance of health motives was associated with a lower level of PA among boys. To get in good shape, to look good and to control weight are aspects of health motives and might be related to body image, which surprisingly starts to be a more important barrier to PA for boys than for girls. Boys are more prone than girls to be physically inactive when they are dissatisfied with their body image [24]. 

Our findings show that social motives are rated higher by girls than by boys and are only associated with PA among girls, which is in line with findings of Wold and Kannas [21] regarding Finnish, Norwegian and Swedish adolescents, and of Litt *et al*. [34] regarding US adolescents. However, Gillison *et al*. [20] reported that boys in the UK are more commonly involved in exercising due to social pressure or to attain ego enhancement, which might be perceived as social motives, though the latter in particular may also be interpreted as being mostly very personal. To have fun, to make new friends, to see friends, to be cool or to have an exciting experience are not just rated higher by girls but are also associated with higher engagement in PA in girls, but not in boys, which was also confirmed by Iannotti *et al*. [13]. These findings are also consistent with the SDT [8] showing that one of the motivators for adolescent PA is enjoyment of the activity. The gender difference in the association of social motives with PA may also have consequences for the design of interventions. In particular for girls, social motives are associated with PA, so developing and stressing that component in interventions to promote PA may attract them more than boys.

This study has several important strengths, the most important being the large and representative samples of adolescents and the high response rates. On the other hand, caution in interpreting our results should reflect the limitations of the self-reported measures and cross-sectional design. We used subjective self-reports for measuring the level of PA. Anonymity, confidentiality and also privacy were provided by self-administration of questionnaires in the absence of teachers; this decreased the probability of the over- or under-reporting of health-related behavior [35]. A review of Brener *et al*. [35] reported moderate to high reliability of self-reported PA, supporting the validity of our data. Furthermore, the cross-sectional design of our study makes it impossible to make conclusive inferences about causality based on our findings. Our findings therefore need to be confirmed in studies with a longitudinal design. 

Significant associations between motives for PA and the level of PA in our findings underline the importance of such motives in adolescents (the higher the score, the more important motive). In order to increase the efficiency and successful implementation of the developed programs for promotion of PA, they need integrate specific motives for PA. Our results indicate that associations between motives and the level of PA partially differ by gender. We found that “Good child” motives and achievement motives are associated with the level of PA among boys, and also among girls. Therefore, one should be aware of different motivations for engagement in PA and prepare interventions in line with this. Focusing on the “Good child” motives and achievement motives in planned programs for the promotion of PA will fit the needs of both girls and boys and might increase their engagement in physical activity. At the same time working with social motives might be effective only among girls, while working with health motives might be effective only among boys, as associations of these motives with physical activity differ with gender according to our results. 

We found cross-sectional associations between the motives for PA and the level of PA, which should preferably be confirmed in longitudinal research in future to establish that the motives for PA cause the level of PA and further be explored in qualitative research regarding the underlying mechanisms. Efforts to increase PA in childhood and adolescence need to determine which motives are effective for the particular population being targeted. However, more research on this topic needs to be undertaken before the association between motives for PA and PA among adolescents is more clearly understood, especially through understanding of other possible variables influencing motives for PA.

## 4. Conclusions

When policy makers and practitioners are planning public health strategies and interventions they should take into account the motives that are more likely associated with a high level of PA. The associations between motives for PA and level of PA partially differ by gender. These gender differences regarding the relations between the four motives (social, health, “Good child” and achievement motives) for PA and engagement in PA among adolescents should be considered in interventions focusing on enhancement of PA. 
